# Therapeutic efficacy and safety of artemether-lumefantrine for uncomplicated *Plasmodium falciparum* malaria treatment in Metehara, Central-east Ethiopia

**DOI:** 10.1186/s12936-024-04991-2

**Published:** 2024-06-12

**Authors:** Mahelet Tesfaye, Ashenafi Assefa, Henok Hailgiorgis, Bokretsion Gidey, Hussein Mohammed, Getachew Tollera, Geremew Tasew, Gudissa Assefa, Worku Bekele, Hassen Mamo

**Affiliations:** 1 Department of Microbial, Cellular and Molecular Biology, College of Natural and Computational Sciences, Addis Ababa University, Addis Ababa, Ethiopia; 2 Ethiopian Public Health Institute, Addis Ababa, Ethiopia; 3 Minstry of Health, Addis Ababa, Ethiopia; 4 World Health Organization, Addis Ababa, Ethiopia; 5 Institute of Infectious Disease and Global Health, University of North Carolina, Chapel Hill, USA

**Keywords:** *Plasmodium falciparum*, Therapeutic efficacy, Artemether-lumefantrine, Metehara

## Abstract

**Background:**

Malaria remains a major global health problem although there was a remarkable achievement between 2000 and 2015. Malaria drug resistance, along with several other factors, presents a significant challenge to malaria control and elimination efforts. Numerous countries in sub-Saharan Africa have documented the presence of confirmed or potential markers of partial resistance against artemisinin, the drug of choice for the treatment of uncomplicated *Plasmodium falciparum* malaria. The World Health Organization (WHO) recommends regular surveillance of artemisinin therapeutic efficacy to inform policy decisions.

**Methods:**

This study aimed to evaluate the therapeutic efficacy of artemether-lumefantrine (AL), which is the first-line treatment for uncomplicated *P. falciparum* malaria in Ethiopia since 2004. Using a single-arm prospective evaluation design, the study assessed the clinical and parasitological responses of patients with uncomplicated *P. falciparum* malaria in Metehara Health Centre, central-east Ethiopia. Out of 2332 malaria suspects (1187 males, 1145 females) screened, 80 (50 males, 30 females) were enrolled, followed up for 28 days, and 73 (44 males, 29 females) completed the follow up. The study was conducted and data was analysed by employing the per-protocol and Kaplan–Meier analyses following the WHO Malaria Therapeutic Efficacy Evaluation Guidelines 2009.

**Results:**

The results indicated rapid parasite clearance and resolution of clinical symptoms, with all patients achieving complete recovery from asexual parasitaemia and fever by day (D) 3. The prevalence of gametocytes decreased from 6.3% on D0 to 2.5% on D2, D3, D7, and ultimately achieving complete clearance afterward.

**Conclusion:**

The overall cure rate for AL treatment was 100%, demonstrating its high efficacy in effectively eliminating malaria parasites in patients. No serious adverse events related to AL treatment were reported during the study, suggesting its safety and tolerability among the participants. These findings confirm that AL remains a highly efficacious treatment for uncomplicated *P. falciparum* malaria in the study site after 20 years of its introduction in Ethiopia.

## Background

Globally, there were estimated 249 million cases and 608,000 deaths attributed to malaria in 2022 [1]. These figures indicate an increase compared to the respective numbers 247 million and 619,000 in 2021, following the disruption caused by the COVID-19 pandemic [2]. Low access to health service in malaria endemic countries combined with socioeconomic factors such as unemployment, low income, and inadequate housing construction [3], human behavioural factors [4] and mosquito insecticide resistance [5], pose significant barriers to effectively mitigating malaria. Additionally, the interplay of land-cover, land-use, urbanization and climate change has contributed to an increased risk of malaria in sub-Saharan Africa [6–11]. These authors argue that, environmental changes resulting from land-cover, land-use, urbanization and/or climate change result in spatial and temporal variations in temperature, humidity, and precipitation, all of which influence the ecology and biology of the vectors and increase the risk of malaria transmission.

Case management using artemisinin-based combination therapy (ACT) is crucial for malaria control and elimination [12]. The World Health Organization (WHO) currently recommends six artemisinin-based combinations for the treatment of uncomplicated *P. falciparum* malaria: artemether-lumefantrine (AL), artesunate-amodiaquine (AS + AQ), artesunate-mefloquine (AS + MQ), artesunate-sulfadoxine-pyrimethamine (AS + SP), dihydroartemisinin-piperaquine (DHA + P), and artesunate-pyronaridine (AS + PY) [13]. AL or AS + AQ are the most widely used artemisinin-based combinations in Africa, while intravenous artesunate, followed by a full course of ACT, is the standard treatment for severe malaria [13].

However, the emergence and rapid spread of drug-resistant strains of the parasite present a significant challenge to malaria control and elimination plan. Resistance to AL has been observed in Southeast Asia [14]. Several countries in sub-Saharan Africa have reported confirmed or potential markers of partial resistance mutations against artemisinin. These countries include Rwanda [15–18], Tanzania [19], Uganda [20–23], Eritrea [24], Ethiopia [25–27], Sudan [28], South Sudan [29], Somalia [30], Ghana [31–33], and the Democratic Republic of Congo [34]. The status of drug resistance markers in Africa has been extensively discussed in various review articles [35–40]. Although there is limited data on treatment failure using AL or AS + AQ in Africa thus far, the increasing evidence pointing to the emergence of validated or candidate resistance markers [40] is highly concerning.

The WHO recommends monitoring the efficacy of anti-malarial treatments regularly to combat drug resistance, following its standard protocol [41]. Regular malaria efficacy testing provides valuable data for updating and refining treatment guidelines [42, 43]. It helps determine which anti-malarial drugs are most effective in specific regions and against certain strains of the malaria parasite. This information enables healthcare professionals to make informed decisions about which drugs to prescribe and helps ensure that patients receive the most appropriate and effective treatment [44, 45]. In line with the global principle, Ethiopia has been actively monitoring the efficacy of malaria drugs through nationwide sentinel sites and using the findings to inform policy decisions. For example, a study conducted in 1997–1998 at 18 sites evaluated the efficacy of chloroquine for treating uncomplicated *P. falciparum* malaria. The results of this study prompted the decision to replace chloroquine with sulfadoxine-pyrimethamine (SP) [46]. Similarly, an efficacy study conducted in 2003 at 10 sites examined the effectiveness of SP, leading to its replacement with AL in July 2004 (reviewed in ref [46]).

Ethiopia has developed a strategic plan for malaria from 2021 to 2025, which has been extended to 2030, and is currently working towards reviewing its malaria program and making progress towards eliminating malaria in areas with low transmission rates [47]. Nationwide surveillance to assess the efficacy of AL for treating malaria is part of this strategic plan. The objective of this study is to evaluate AL therapeutic efficacy in Metehara, which is one of the multiple sentinel sites in the country, for the treatment of uncomplicated *P. falciparum* malaria.

## Methods

### Study site

Metehara is a town located in the Oromia Region of Ethiopia, situated about 200 km southeast of Addis Ababa. Its geographic coordinates are latitude and longitude of 08°54′N 39°55′E with an elevation of 947 m above sea level. It is positioned along the main highway that connects Addis Ababa with the eastern parts of the country, making it an important transportation hub. Geographically, Metehara is situated in the Great Rift Valley, which is a geological trench that extends from the Middle East to Mozambique. Vast plains, scattered hills, and volcanic formations characterize the surrounding landscape. In terms of climate, Metehara experiences a semi-arid climate with hot and dry conditions. The town lies in a lowland region, resulting in high temperatures throughout the year. According to the local areas unpublished agro-ecological data, the average annual temperature ranges from 24 to 30 °C. The hottest months are typically from March to May, with temperatures often exceeding 35 °C. The coolest months are from November to February, with average temperatures of 18–24 °C.

The town’s meteorological data show that Metehara receives most of its rainfall during the wet season, which typically occurs from June to September. The average annual precipitation is around 600–800 mm. The vegetation in and around Metehara is mainly characterized by savannah grasslands and scattered acacia trees. Metehara is also known for its proximity to the Awash National Park, which is located about 50 km east of the town. Overall, Metehara's location in the Great Rift Valley, coupled with its semi-arid climate and diverse wildlife, makes it an interesting and unique area in Ethiopia. Based on the Ethiopian Central Statistical Agency Population Projection of 2014 [48], the town is inhabited by 28,746 people.

Nearby waterbodies that characterizes the town are the Awash River and Lake Beseq’a. The presence of these water sources, along with the irrigation system of the Metehara sugar factory that relies on the Awash River, creates a favourable microhabitat for breeding malaria vectors. Additionally, the low altitude of Metehara town places it in a region classified as a ‘high malaria’ endemic area [47]. Numerous studies conducted in Metehara town and its surroundings [49–56], as well as the town's health record system, indicate that malaria persists throughout the year but varies in intensity. The primary transmission occurs from September to November, with a minor peak from March to May. According to unpublished data from Metehara town health records, *P. falciparum* infections account for 63% of clinical malaria cases in Metehara.

Recently, there has been a resurgence of malaria in the town [57], possibly associated with the emergence and spread of the Asian urban malaria vector *Anopheles stephensi* [58]. Although published studies, Ministry of Health reports, and local health system reports agree that malaria in Metehara has historically been predominantly attributed to *P. falciparum*, a recent community-based study [59], which was conducted during the dry season targeted asymptomatic cases using highly sensitive molecular tools, demonstrated the predominance of *Plasmodium vivax*, at least in the study community and season.

### Study design

The study was conducted from November 2020 to March 2021. It employed a single-arm prospective evaluation design, wherein patients who visited the outpatient department of the Metehara Health Centre (MHC) and who received partially supervised AL treatment were monitored for 28 consecutive days. Therapeutic responses on day (D) 28 were the study primary outcomes (endpoints), and are classified as either adequate clinical and parasitological response (ACPR), or treatment failure (TF); designated as early treatment failure (ETF), late clinical failure (LCF), or late parasitological failure (LPF). The secondary outcomes (endpoints) were parasite clearance rate (proportion of patients with negative blood smears on D1, D2 and D3), fever clearance rate (proportion of patients without fever on D1, D2 and D3), gametocyte carriage rate (proportion of patients with gametocytes during the 28 day follow-up period).

### Study population and inclusion/exclusion criteria

The study included patients who met the inclusion criteria established by the WHO for evaluating and monitoring the efficacy of anti-malarial drugs [13]. These include the presence of fever (axillary temperature ≥ 37.5 °C) or a history of fever the previous 24 h, bodyweight greater than 5 kg, age over 6 months, microscopically-confirmed *P. falciparum* mono-infection with parasitaemia ranging from 1000 to 200,000 asexual parasites per microlitre (μL) of blood, the ability to swallow oral medication, permanent residency within the health centre's catchment area, and willingness to comply with the study protocol.

Exclusion criteria comprised severe malaria, infection with mixed or non-falciparum species, severe malnutrition, other febrile conditions, haemoglobin (Hb) level below 5.0 g/dL, intake of AL within the previous 2 weeks, inability to take oral medication or experiencing continuous vomiting, known chronic or severe diseases, hypersensitivity to the study drugs, and pregnancy or breastfeeding.

### Sample size determination

A minimum of 73 sample size was determined using the formula *n* = *(Z*^2^*p*(1 − p))/(d^*2*^*)* [60], where: ‘*n*’ is the required sample size, ‘*Z*’ is the *Z*-score corresponding to the desired confidence interval (CI) (*Z* = 1.96 for a 95% CI), ‘p’ is the estimated treatment failure rate (5%), ‘d’ is the desired level of precision (5%). To account for potential loss-to-follow-up (LTFU), withdrawals, or exclusions due to reinfection with PCR correction, an additional 20% of patients is added [61]. This would result in a total enrollment requirement of 88 patients. However, due to logistical reasons, the study was unable to reach the desired sample size and recruited only 80 patients.

### Baseline screening

For each patient, socio-demographic and clinical data were recorded. Baseline measurements of axillary temperature and bodyweight were taken. Finger-prick blood samples were used to prepare thick and thin smears, which were then stained with 10% Giemsa following standard procedures [62]. Expert microscopists examined the slides to detect malaria parasites, identify the species, quantify parasitaemia, and determine gametocyte carriage. These assessments were conducted at baseline and during all scheduled and unscheduled visits throughout the 28-day follow-up period.

### Enrollment and follow-up

Patients who met the inclusion criteria were enrolled and initiated an oral treatment with AL. The AL used (20 mg + 120 mg, batch no: (10): HWE110049; Mfg: (11), 01/2020; Exp.: (17):12/2021, *Ipca Laboratories Ltd*, Maharashtra, (India)) was provided by the Ethiopian Ministry of Health through WHO support. The drug dosage was determined based on the revised weight-based guidelines provided by the WHO [13]. The treatment involves administering AL twice daily for a period of three days, totaling six doses. The initial dose is given at 0 h on D1, followed by a second dose between 8 to 12 h later. Subsequent doses on D2 and D3 were administered twice daily, in the morning and evening. For individuals weighing 5 to < 15 kg, 15 to < 25 kg, 25 to < 35 kg, and ≥ 35 kg, 1, 2, 3, and 4 tablets of the 20 mg + 120 mg combination were provided, respectively, for each dose. For young children, the medication was crushed, mixed with water, and administered as a suspension. Older children and adults, on the other hand, took tablets orally with a glass of water. During each supervised drug administration, patients were closely monitored for a period of 30 min. In the event of vomiting occurring before the 30 min mark, the dose was repeated, and the patient was observed for an additional 30 min.

The initial dose and all morning doses were given at the health centre under the direct supervision of the study team. To ensure proper administration of the medication at home, patients and/or parents/guardians were given clear instructions regarding the evening doses. Patients or caregivers were instructed to return to the health centre if the patient vomited the medication administered at home. To ensure continuous care, a nurse was assigned to care for the study patients at the health centre during night or off-duty hours. During subsequent visits on D1, D2, and D3, the success of drug administration at home was assessed. Scheduled follow-up visits were on D7, D14, D21, and D28, with additional unscheduled visits for participants who felt unwell.

### Assessment of adverse events

Adverse events (AEs) were evaluated through clinical and physical examinations, and questioning using a standard list of AEs associated with malaria and AL. Caregivers were asked to report any unusual occurrences after drug administration, including the child's tolerability to the treatment.

### Data analysis

The therapeutic efficacy, sociodemographic and clinical data were double entered into the WHO Excel spreadsheet specifically designed for this purpose. Descriptive statistics were employed to summarize the continuous variables, and the Student's t-test was conducted to compare these variables between groups. Statistical significance was set at a p-value < 0.05. For the analysis of survival data (primary endpoint), the standard WHO Per-protocol (PP) analysis and Kaplan–Meier (K–M) survival estimator were utilized.

Qualitative sociodemographic data including information on the history of clinical malaria, possession of a bed net, and reported regular utilization of the bed net, were consolidated into a table along with quantitative data. Clinical qualitative data encompassed self-reported symptoms, such as fever, headache, nausea, and dizziness, without indications of general danger or severe *P. falciparum* malaria. AEs reported by the participants were summarized descriptively and tabulated as per the WHO’s guidelines on the pharmacovigilance of anti-malarial medicines [63]. However, the nature of the events, their severity, duration, and any pertinent contextual information were discussed individually with the patients. Additionally, instances of LTFU and involuntary protocol violations were counted recorded. Overall, the study utilized a combination of qualitative and quantitative data collection approaches, as well as descriptive summaries and individual case studies, in order to provide a holistic understanding of the sociodemographic factors, clinical data, AEs, and study compliance within the participants.

## Results

### Population screened

A total of 2332 individuals (1187 males and 1145 females) suspected of having malaria visited MHC for malaria diagnosis. Among them, 178 (7.6%) tested positive for malaria. Out of these 178 positive cases, 102 (57.3%) were initially identified as *P. falciparum* mono-infections, while 76 (42.7%) were identified as *P. vivax* infections.

### Patients’ characteristics at enrollment

Among the 102 individuals with *P. falciparum* infections, 89 were eligible for the study, but 9 could not participate due to their mobile working arrangements. Therefore, at baseline (D0), there were 80 patients, 50 males and 30 females. The mean age of the population was 17.7 ± 14.24, with 15 under-five, 26 between 5–15 years and 39 over 15. The minimum age was 8 months and the maximum 65 years. While the overall mean bodyweight was 36.8 ± 19.9 kg, it was 10.9 ± 3.08, 23.1 ± 6.72, 55.4 ± 9.75 for the < 5, 5–15 and > 15 age groups respectively. Twenty-nine (36.3%) participants reported a previous history of confirmed malaria, and 38 (47.5%) reported the availability of a bed net, but only 27 (33.8%) affirmed that they were using it regularly.

At the baseline, individuals with body temperature of  ≥ 37.5 °C were 29 (36.3%) and the rest had self-reported fever in the past 24 h. The mean baseline body temperatures were 37.7 ± 1.3 for the under-five, 37.7 ± 1.34 for 5–15, and 35.8 ± 5.9 for over 15 with overall mean of 36.8 °C. The range of body temperature was 32.5–40.2 °C. The mean baseline Hb level was 8.3 ± 2.16 g/dL, 9.7 ± 2.0 g/dL and 11.4 ± 1.5 g/dL for the < 5, 5–15 and > 15 age groups respectively, and the overall mean Hb level was 10.41 ± 2.1. During baseline, 42 (52.5%) patients were anaemic (14 (33.3%) severe (Hb < 8 g/dL), 16 (38.1%) moderate (Hb 8–10 g/dL), and 12 (28.5%) mild (Hb 10 g/dL). Of these, 23(54.8%) were male and 19 (45.2%) were female. The highest prevalence of anaemia (40.5%) was recorded among the age group 5–15 years, 31.0% among < 5-year old and 28.5% among > 15 years of age.

Baseline geometric mean parasite density (GMPD) was 10,627 ± 20,736.7 asexual parasites/μL of blood. There were 41 patients having parasitaemia ≥ 10,000 parasites/µl and 39 patients had parasitaemia 1000–9999 parasites/µL. There was a significant variation in GMPD parasitaemia between children < 5 years old (3,754.62 ± 4,435.472 parasites/μL) and those 5–15 years old (14,278.08 ± 27,981.34 parasites/μL). Three patients had a parasitaemia level exceeding 50,000 parasites/μL, maximum 98,235 parasites/μL. The gametocyte prevalence at baseline was 6.3% (5/80), with gametocyte numbers ranging from 80 to 5320 sexual parasites/μL of blood (Table 1).

**Table 1:** Mean or number (proportion) of baseline characteristics of the study participants at Metehara Health Centre, Central-east Ethiopia

Variables	Age category			
	< 5 (n = 15)	5–15 (n = 26)	> 15 (n = 39)	Overall (n = 80)
Age, years	3 ± 1.2	9 ± 2.24	30 ± 11.9	18 ± 14.6
Gender				
Male, no. (%)	8 (53.3)	16 (61.5)	26 (66.7)	50 (62.5)
Female, no. (%)	7 (46.7)	10 (38.5)	13 (33.3)	30 (37.5)
Body temp., ºC ± SD	37.7 ± 1.3	37.7 ± 1.38	36.7 ± 0.92	37.2 ± 1.25
Bodyweight ± SD	10.9 ± 3.08	23.1 ± 6.72	55.4 ± 9.75	36.8 ± 19.9
Hb, g/dL ± SD	8.6 ± 2.18	9.88 ± 2.01	11.6 ± 1.42	10.5 ± 2.14
Parasitemia per μL ± SD	3754.62 ± 4435.472	14,278.08 ± 27,981.343	10,679.03 ± 18,193.26	10,627 ± 20,736.7
Gametocyte pos., no. (%)	1 (7.7)	3 (12.6)	2 (5.8)	6 (8.4)
Bed net use, no. (%)				
Yes, no. (%)	5 (33.3)	7 (26.9)	19 (48.7)	30 (37.5)
No, no. (%)	10 (66.7)	19 (73.1)	20 (51.3)	50 (62.5)
Past malaria, no. (%)				
Yes, no. (%)	4 (26.7)	9 (34.6)	21 (53.8)	34 (42.5)
No, no. (%)	11 (73.3)	17 (65.4)	18 (46.2)	46 (57.5)

*no*. number, % per cent, *ºC* degree Celsius, *SD* standard deviation, *g/dL* gram per deciliter

### Protocol violations including LTFU

The baseline participant number was reduced to 79, 78, 77, 74, and 73, respectively, on D1, D7, D14, D21, D28. This was because there was one LTFU on each of the days. All of the LTFU were males and their ages of those lost on days 1, 7, 14, 21 and 28 were 28, 32, 28, 10 and 29 years, respectively. Additionally, there were two cases of mixed infection with *P. vivax* (involuntary protocol violations) on D21, a 5-year girl and a 50-year man.

### Primary endpoint

**Fig. 1:**
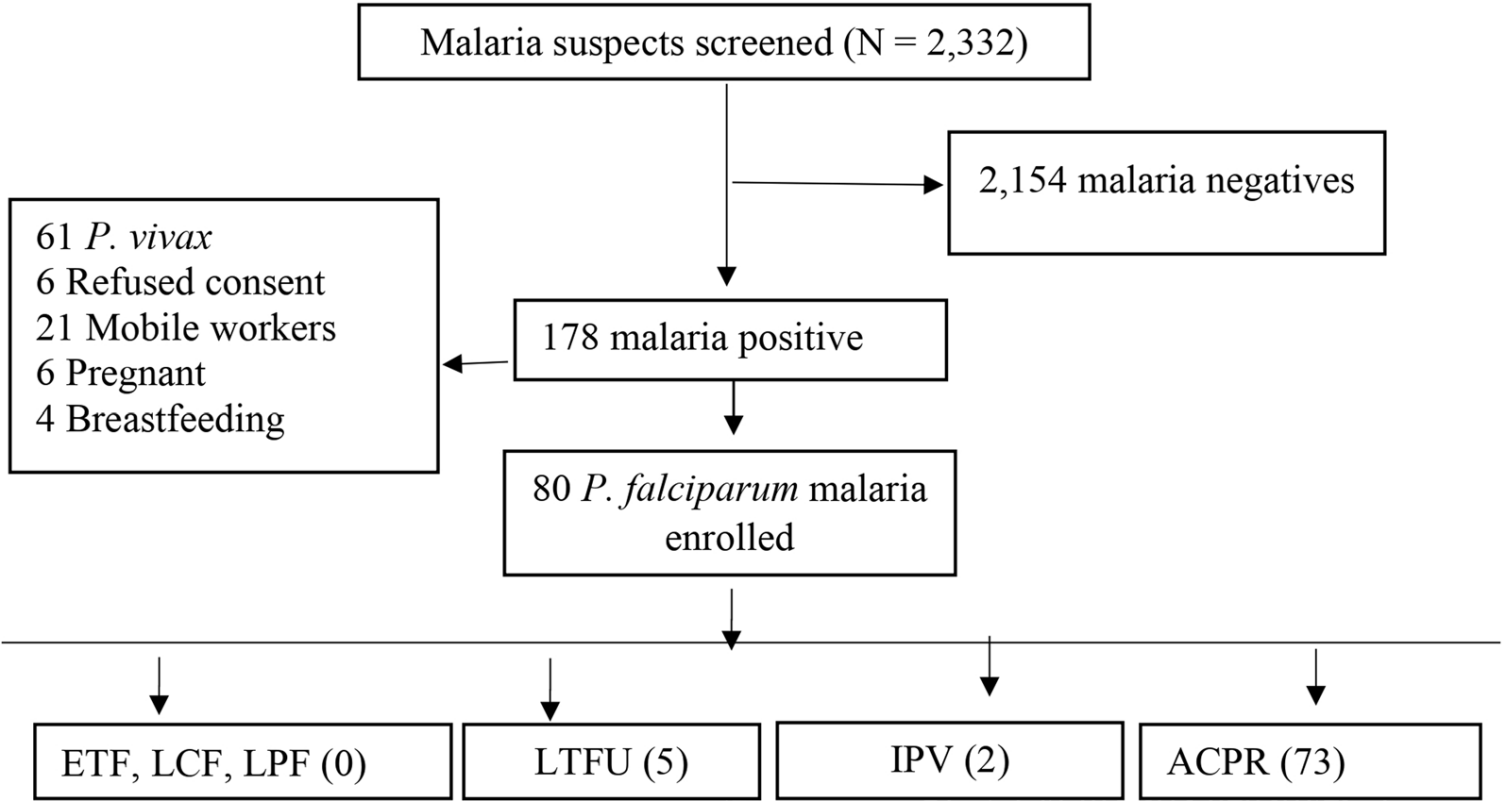
Screening, enrollment, follow-up and treatment outcomes of *P. falciparum* malaria patients in Metehara Health Centre, Central-east Ethiopia. *ACPR* adequate clinical and parasitological response, *ETF* early treatment failure, *LCF* late clinical failure, *LPF* late parasitological failure, *IPV* involuntary protocol violation, *LTFU* loss-to-follow-up

The cure rate was 100% based on the PP analysis, with no ETF, LCF, or LPF (Fig. 1; Table 2). The PCR-uncorrected cure rate was also 100% according to the K-M analysis. The analysis did not account for PCR-correction for any possible submicroscopic infections due to logistic constraints. The use of PCR helps ensure that all infections, including submicroscopic ones, are detected and accounted for. Overall, a high cure rate was observed in both the PP and the K-M analyses.

**Table 2:** Treatment outcomes based on Per Protocol analysis among patients treated with artemether-lumefantrine at Metehara Health Centre, Central-east Ethiopia

Description	Frequency in each age (year) category			
	< 5 (n = 15)	5–15 (n = 26)	> 15 (n = 39)	Overall
ACPR	14 (93.3%)	25 (96.2%)	34 (87.2%)	73 (100)
ETF	0 (0)	0 (0)	0 (0)	0 (0)
LTF	0 (0)	0 (0)	0 (0)	0 (0)
LPF	0 (0)	0 (0)	0 (0)	0 (0)
LTFU	0 (0)	1 (28.6%)	4 (71.4%)	5 (10.9%)
PV	1	0	1	2
W	0 (0)	0 (0)	0 (0)	0 (0)

*ACPR* adequate clinical and parasitological response, *ETF *early treatment failure, *LTF *late treatment failure, *LPF *late parasitological failure, *LTFU *loss-to-follow-up, *PV *protocol violation, *W *withdrawal

### Secondary endpoints

#### Parasite and fever clearance

At the baseline, 41 (51.3%) of the study participants had a parasitic load of  ≥ 10,000 asexual parasites/μL. Following AL administration, the parasite count rapidly decreased to zero by D2, and subsequently, no asexual parasites were detected. Complete parasite clearance was achieved before D3 and maintained until D28. Upon enrollment (n = 80), 29 (36.3%) individuals had an axillary temperature of  ≥ 37.5 °C. This percentage decreased to 10.1% (8 out of 79) on D1, reached zero on D2, and was at 1.3% (1 out of 79) on D3, taking into account the LTFU of one participant on D1. Fever and parasites both were rapidly eliminated within the initial three days of treatment, with only one individual experiencing fever on D7 in the absence of parasites.

#### Gametocytaemia clearance

At the baseline, gametocytes were detected in 5 participants, representing a prevalence of 6.3%. Among these 5 individuals with gametocytes, 11.5% (3 out of 26) were aged 5–15 years, 5.1% (2 out of 39) were > 15 years, and 6.7% (1 out of 15) were < 5 years of age. The proportion of participants with gametocyte carriage declined from 6.3% on D0 to 2.5% on D2, D3, and D7, with complete disappearance thereafter.

#### Adverse events

At baseline, the patients presented with common signs/symptoms associated with malaria. The most frequently reported symptoms were headaches, experienced by 39 (48.9%) participants followed by nausea 8 (10%). AEs ‘not probably related’ to the medication were observed at different time points after the administration of the AL treatment. On D3 and D7, one report each was recorded. Two signs were reported on D14, and on both D21 and D28, one sign each was reported. It is worth noting that none of these signs and symptoms were serious, and the majority of them resolved as parasitaemia was cleared. No participants withdrew from the study due to AEs, and there were no AEs of special interest (Table 3).

**Table 3:** Adverse events at enrollment and during follow-up among patients in a therapeutic efficacy study of artemether-lumefantrine against uncomplicated *Plasmodium falciparum* malaria, Metehara Health Centre, Central-east Ethiopia

AE	Onset	Duration, days	Seriousness	Relationship to treatment	Frequency no. (%)	Withdrawals	Of special interest
Headache	D0	1	Mild	NA	39 (48.7)	None	Not
Nausea	D0	1	Mild	NA	8 (10.0)	None	Not
Vomiting	D0	1	Mild	NA	1 (2.5)	None	Not
Cough	D3	1	Mild	PNR	1 (2.5)	None	Not
Back pain	D7	1	Mild	PNR	1 (1.3)	None	Not
Dizziness	D14	1	Mild	PNR	2 (2.5)	None	Not
Abdominal pain	D21	1	Mild	PNR	1 (1.3)	None	Not
Diarrhea	D28	1	Mild	PNR	1 (1.3)	None	Not

*AE* adverse even, *NA* not applicable, *PNR* ‘Probably Not Related’, *no.* number of patients, % percentage

## Discussion

In our retrospective assessment of the past six-year duration (2018–2023), there was a continuous increase in malaria prevalence in Metehara. Although *P. falciparum* accounted for most of the cases, the number of *P. vivax* was also sizable. This observation of the recent resurgence of malaria in the study area, along with the relative dominance of *P. falciparum*, corroborates a retrospective study that was previously mentioned [57]. The malaria resurgence could be due to factors such as resource constraints, ineffective preventive measures, and the development of insecticide resistance, and/or the spread of *An. stephensi*. Scaling-up of control interventions and identifying malaria transmission hotspots, along with their corresponding spatiotemporal and anthropogenic risk factors is warranted.

This study demonstrated a 100% cure rate, indicating that all participants who strictly adhered to the study protocol achieved successful treatment outcomes. The overall findings of the study indicate that a standard six-dose treatment of AL achieved a 100% cure rate for uncomplicated *P. falciparum* malaria over a 28-day period, without PCR correction. The presence of parasitaemia on D3 is considered a key indicator for suspected artemisinin resistance [64]. However, in this study, the baseline mean parasitaemia was 10,627 ± 20,736.7, which decreased to zero on D3. Artemether, being a potent anti-malarial drug that is rapidly absorbed, leads to a rapid reduction in parasite biomass, prompt symptomatic improvement, and rapid elimination [65]. Consequently, the absence of ETF confirms the non-existence of artemisinin-resistant *P. falciparum* strains among the study population although this may not be ruled out from the study areas as a whole. Similarly, the study did not observe LTF or LPF. The adjusted cure rate at D28 was found to be 100% for children < 5, as well as for the age groups of 5–15 and over 15.

Despite presenting with high fever and parasitaemia upon enrollment, the administration of the drug effectively eliminated parasitaemia by D2 and led to a decline in fever to within the normal range by D1. No cases of LCF were observed. These findings demonstrate the rapid clearance of parasites, prevention of disease progression, immediate symptom resolution, and reduced risk of complicated malaria associated with the use of AL. It is known that high levels of parasitaemia can contribute to severe fever, as fever is an immune response triggered by the infection and serves as an indication of the parasite's accelerated replication [66]. In contrast to results from Southeast Asian nations where delayed fever clearance was observed with AL, the rapid fever-resolving ability of AL has been consistently observed in efficacy tests conducted in Ethiopia. Meta-analysis of anti-malarial treatment outcomes in Ethiopia reported high efficacy of AL [67–69], which is consistent with the findings of the present study. However, in this study, only 15 participants were aged below 5 years. It is possible that participants above the age of 5 may have already developed anti-malarial immunity, which could potentially lead to an overestimation of the efficacy of the anti-malarial drug AL. Additionally; the relatively lower mean parasitaemia observed in this study (~ 11,000 parasites/µL) may have influenced the efficacy results as well.

AL primarily targets the asexual stage of the malaria parasite to decrease and clear it, but it also exhibits gametocidal activity. The current study confirmed the rapid gametocidal activity of AL, gametocyte carriage at D0 decreased over and disappeared after D7. This activity of the drug interrupts the transmission cycle between the mosquito vector and the human host. A study using membrane-feeding *Anopheles* mosquitoes demonstrated a reduction in malaria transmission following the six-dose regimen of AL [70]. The authors reported that gametocyte clearance was observed by D2, with one case persisting until D3 and completely disappearing by D7 and onwards. However, it is understood that AL is strongly active against younger gametocytes, which is why the Ethiopian falciparum treatment guidelines includes single low-dose primaquine to target mature gametocyte stages.

Malaria has the potential to cause anaemia, which is characterized by a decrease in Hb levels. The effect of malaria treatments on Hb levels may vary. Some studies have shown that effective treatment of malaria with anti-malarial drugs helps clear the infection, allowing the body to recover and restore Hb levels [71, 72]. On the other hand, anti-malarial treatments are associated with a decrease in Hb levels or exacerbated anaemia in some individuals with severe malaria or HIV infection [73, 74]. Moreover, the medication primaquine, used for the radical cure of *P. vivax malaria* and against *P. falciparum* gametocytes, may cause haemolysis, leading to a drop in Hb levels [75, 76].

No serious adverse events were noted, and the majority of the reported reactions were already recognized by the manufacturer as common adverse reactions and documented with the Food and Drug Administration. Once the parasites were cleared, these minor symptoms quickly resolved except one or incidents around the end of the study whose causes could not be established. The results of other studies in Ethiopia [51, 77] align with the absence of serious AE following AL treatment in the current investigation.

Nevertheless, this study has certain limitations. In fact, some of these limitations are limitations of most other similar malaria drug efficacy studies. The study focuses on a specific study area, and the findings may not be representative of the entire region or other geographical locations. The prevalence of malaria and the effectiveness of treatment may vary in different settings. The study's duration was 28 days. Assessing the sustainability of the observed trends and treatment efficacy over a longer period would provide conclusions that are more robust. Sample size appears the minimum and the method of participant selection is nonrandom, it is convenience sampling. A small sample size or biased selection process could affect the generalizability and validity of the study's findings. Potential confounding factors that could influence the study outcomes are not rigorously considered. Factors such as socio-economic status, access to healthcare, and individual behaviors might have influenced the prevalence of malaria and treatment outcomes.

Besides, while the study documented no serious AEs, a more comprehensive information on AEs or their frequency was not obtainable. A more detailed analysis of AEs would provide a better understanding of the safety profile of the treatment. Furthermore, the study did not utilize molecular analysis to confirm the absence of artemisinin-resistant *P. falciparum* strains. Molecular analysis is crucial for detecting resistance markers and assessing the potential spread of resistant strains. Financial limitations prevented the provision of fatty food alongside AL administration. This may have influenced the drug's bioavailability and potentially affected treatment outcomes, although there was no phenotypic treatment failure and microscopically, highlighting a limitation in the study design.

Moreover, the study did not measure drug blood concentration, and did not use PCR to detect submicroscopic low-level parasitaemia. One of the key aspects in assessing the efficacy of a malaria drug is determining the concentration of the drug in the bloodstream. Measuring drug blood concentration helps to understand how much of the drug is present in the body, which can directly influence its effectiveness in eliminating the malaria parasite. Without measuring drug blood concentration, it becomes difficult to establish a clear relationship between drug dosage and treatment outcomes. Additionally, variations in drug metabolism or drug interactions may affect drug blood concentration, and without this measurement, it is challenging to determine the reasons behind treatment failures or successes accurately [78–80].

This study has further limitations. Hb levels were not measured, because of logistic problem, during the follow-up period. This made it impossible to determine the participants' anemia status and assess the potential impact of the treatment on this parameter. Additionally, the exclusion of seven participants during the follow-up period resulted in a reduction in the sample size, which is just the minimum threshold of 73 participants set by the WHO. This reduction in sample size may have affected the statistical power and generalizability of the study findings. These limitations underscore the need for improvements in adherence to the study protocol and the inclusion of a larger sample size from the outset. Addressing these issues, in future studies will enhance the validity, reliability, and generalizability of findings in this area.

## Conclusions

Notwithstanding its limitations, the study revealed that the administration of AL resulted in rapid clearance of parasitaemia and a decline in fever, indicating that the drug effectively clears parasites, prevents disease progression, and resolves symptoms quickly some 20 years after its introduction in Ethiopia. AL also exhibited gametocidal activity, which interrupts the transmission cycle of malaria although its activity against mature gametocytes appears limited. Overall, the study demonstrated the high efficacy of AL in the study area during the study period and can serve as a valuable source of information for policy decision.

## Data Availability

All data generated and analysed in the study are included in this manuscript.

## Abbreviations

ACPR: Adequate clinical and parasitological response
ACT: Artemisinin-based combination therapy
AEs: Adverse events
AL: Artemether-lumefantrine
AS+AQ: Artesunate-amodiaquine
AS+MQ: Artesunate-mefloquine
AS+SP: Artesunate-sulfadoxine-pyrimethamine
AS+PY: Artesunate-pyronaridine
CI: Confidence interval
COVID: Coronavirus disease-2019
D: Day
DHA+P: Dihydroartemisinin-piperaquine
ETF: Early treatment failure
FMoH: Federal Ministry of Health
GMPD: Geometric mean parasite density
g/dL: Gram per deciliter
Hb: Haemoglobin
h: Hour
kg: Kilogram
IRB: Institutional review board
K–M: Kaplan–Meier
LCF: Late clinical failure
LPF: Late parasitological failure
LTFU: Loss-to-follow-up
µL: Microliter
mg: Milligram
MHC: Metehara Health Centre
PP: Per-protocol
PCR: Polymerase chain reaction
PV: Protocol violation
SP: Sulfadoxine-pyrimethamine
WHO: World Health Organization
